# The Treatment of Skin Cancers

**Published:** 1898-11

**Authors:** 


### Book Reviews.

THE TREATMENT OF SKIN CANCERS. By W. S. GOTTHEIL, M. D., Professor of Dermatology at the New York School of Clinical Medicine; Dermatologist to the Lebanon Hospital, the Northwestern and West-side German Dispensaries, etc. New York: Published by the International Journal of Surgery Company, 100 William Street.

This little brochure of 67 pages, by Gottheil, which treats especially of the diagnosis and treatment of skin cancers, while the etiology and pathology are but briefly considered, will well repay the half hour required for its perusal. Considerable space is devoted to describing the various methods and the different caustics recommended for the destruction of this malignant process, and the author concludes that arsenious acid, owing to its pacific selective action, is the safest, surest and best remedy for this purpose. We cannot better express our appreciation of the book than to quote the last sentence in Dr. Gottheil's preface: "It is my hope that what I have written may do something toward taking this most eligible method of treating cutaneous carcinomata out of the hands of unauthorised practitioners, and placing it where it belongs, at the disposal of the profession at large." E. W.

